# Evidence-Based Approaches to Anticoagulation in Reconstructive Microsurgery—A Systematic Literature Review

**DOI:** 10.3390/life14010082

**Published:** 2024-01-03

**Authors:** Niklas Biermann, Juy Chi Chak, Anna Wiesmeier, Silvan M. Klein, Marc Ruewe, Steffen Spoerl, Philipp Kruppa, Lukas Prantl, Alexandra M. Anker

**Affiliations:** 1 Department of Plastic, Hand- and Reconstructive Surgery, University Hospital Regensburg, Franz-Josef-Strauß-Allee 11, D-93053 Regensburg, Germany; niklas.biermann@ukr.de (N.B.); juy-chi.chak@ukr.de (J.C.C.); anna.wiesmeier@ukr.de (A.W.); silvan.klein@ukr.de (S.M.K.); lukas.prantl@ukr.de (L.P.); 2 Clinic and Polyclinic for Oral and Maxillofacial Surgery, University Hospital Regensburg, Franz-Josef-Strauß-Allee 11, D-93053 Regensburg, Germany; steffen.spoerl@ukr.de; 3 Department of Plastic, Hand- and Reconstructive Surgery, Ernst von Bergmann Klinikum Potsdam, Charlottenstraße 72, D-14467 Potsdam, Germany; kruppaph@gmail.com

**Keywords:** systematic review, anticoagulants, microsurgery, free flaps, flap survival, replantation surgery, thrombosis prevention, aspirin, heparin, dalteparin

## Abstract

This systematic review addresses the crucial role of anticoagulation in microsurgical procedures, focusing on free flap reconstruction and replantation surgeries. The objective was to balance the prevention of thrombotic complications commonly leading to flap failure, with the risk of increased bleeding complications associated with anticoagulant use. A meticulous PubMed literature search following Evidence-Based-Practice principles yielded 79 relevant articles, including both clinical and animal studies. The full-texts were carefully reviewed and evaluated by the modified Coleman methodology score. Clinical studies revealed diverse perioperative regimens, primarily based on aspirin, heparin, and dextran. Meta-analyses demonstrated similar flap loss rates with heparin or aspirin. High doses of dalteparin or heparin, however, correlated with higher flap loss rates than low dose administration. Use of dextran is not recommended due to severe systemic complications. In animal studies, systemic heparin administration showed predominantly favorable results, while topical application and intraluminal irrigation consistently exhibited significant benefits in flap survival. The insights from this conducted systematic review serve as a foundational pillar towards the establishment of evidence-based guidelines for anticoagulation in microsurgery. An average Coleman score of 55 (maximum 103), indicating low overall study quality, however, emphasizes the need for large multi-institutional, randomized-clinical trials as the next vital step.

## 1. Introduction

Anticoagulation represents a critical element in the management of patients undergoing microsurgical procedures in plastic surgery, including free flap and replantation surgeries. Its primary objective is to prevent thromboembolic complications, which still are the leading cause of flap failure and compromise the overall success of the surgical procedure [1]. On the other hand, extensive use of anticoagulative agents may also increase the risk of bleeding complications, thereby potentially compromising flap viability and overall patient outcome [2].

Despite the paramount importance of anticoagulation in microsurgery, only limited consensus has been reached among plastic surgeons regarding the optimal approach to its administration [3,4,5]. Substantial variation exists in terms of the choice of anticoagulants (e.g., heparin), antiplatelet substances (e.g., aspirin) or other drugs influencing rheologic behavior (e.g., dextran), the appropriate concentration (e.g., prophylactic, therapeutic dosage), as well as the optimal timing for administration [3,5,6].

In recent years, a multitude of studies have been conducted to investigate the use of anticoagulative agents in microsurgical procedures, aiming to improve therapeutic strategies and minimize postoperative complications [7,8,9,10,11].

Accordingly, the objective of this systematic review is to search the existing body of the literature on anticoagulation in microsurgery, with a specific focus on free flap and replantation surgery, to provide an updated and comprehensive overview of an evidence-based approach to anticoagulation, ultimately leading to improved patient outcomes and enhanced surgical success.

## 2. Materials and Methods

This literature research was performed respecting the principles of Evidence-Based-Practice (EBP). Valid guidelines of national and international standards were applied (“Guidelines International Network” (http://www.g-i-n.net/ (accessed on 22 November 2022)) and “National Guideline Clearinghouse” (https://www.guideline.gov/ accessed on 22 November 2022)). Following the Preferred Reporting Items for Systematic Reviews and Meta-Analyses (PRISMA), a systematic review of the literature was performed. The PubMed database was searched for meta-analyses, systematic or narrative reviews and primary clinical, animal or laboratory studies on 22 November 2022, using the search string (anticoag* and (free and flap and microsurg* or salvage or microsurg*). Articles in languages other than English or German were excluded. In total, the search yielded 1366 studies. The primary screening process was performed using the abstract and title.

Articles without any abstract or full-text and studies describing case reports, letters to the editor, all states of hypocoagulability and hypercoagulability that were not drug-induced, extracorporeal perfusion or comprising fewer than 10 patients were excluded. A complete overview of inclusion and exclusion criteria is provided in Table 1.

**Figure 1:**
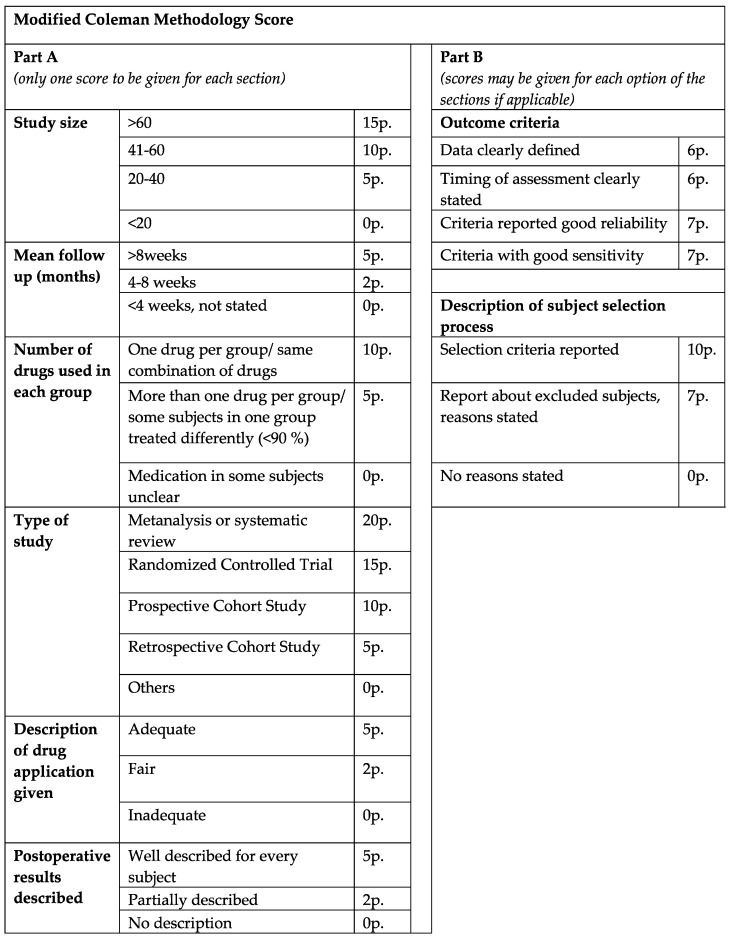
Modified Coleman methodology score. The clinical studies were rated excellent (>80), good (>60), fair (>40) and poor quality (poor ≤ 40), in relation to a maximum score value of 103 points.

The final articles were divided into two main groups, consisting of either clinical or animal studies. To assess the scientific quality of the reviewed clinical studies, a demand-actuated modified version of the Coleman methodology score (CMS) was implemented and utilized. The manuscripts were rated as excellent (>80), good (>60), fair (>40) and poor quality (poor ≤ 40), in relation to a maximum score value of 103 points (Figure 1).

## 3. Results

### 3.1. Article Screening

**Figure 2:**
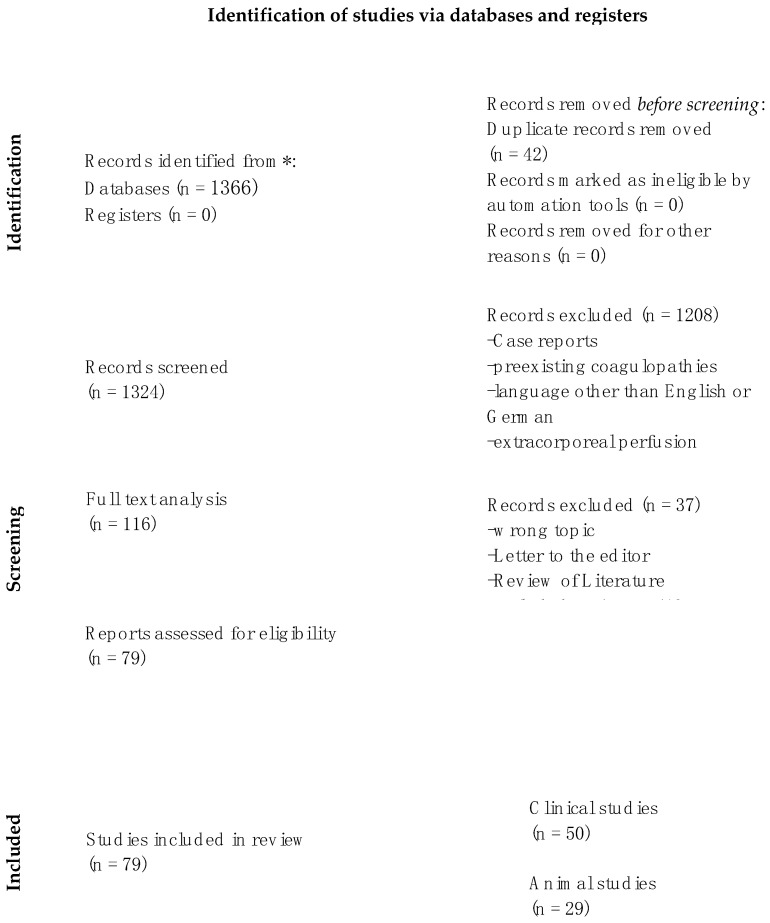
PRISMA flow diagram of the systematic literature review search (adapted from Moher et al. (2009) [12], illustrating the number of studies retrieved from the systematic search and the final number of included studies. * accessed on 22 November 2022.

According to our search terms, 1324 articles were identified and screened after duplicates were removed. Applying our exclusion criteria led to 116 full-texts to analyze. Further excluding small study groups (*n* < 10), non-systematic literature reviews or wrong topics resulted in 79 eligible articles that were included in our investigation. The final article selection was divided into clinical and animal studies (Figure 2). Relevant characteristics of the included studies are summarized in Table S1 (clinical studies) and Table S2 (animal studies).

**Figure 3:**
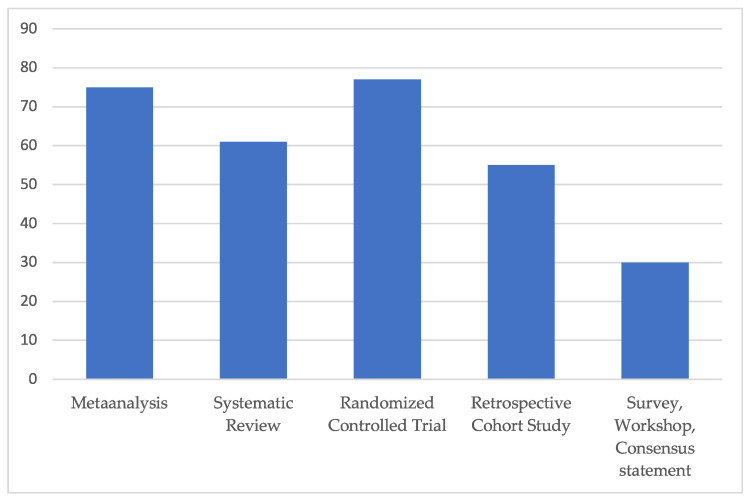
Subgroup analysis of average modified Coleman score for each included study type. All included clinical studies were rated excellent (>80), good (>60), fair (>40) and poor quality (poor ≤ 40), in relation to a maximum score value of 103 points. The average Coleman score rating of all included studies, independent of any study type, accounted for 55, corresponding to “fair” overall study quality.

The average Coleman score rating of all the included clinical studies accounted for 55, corresponding to “fair” overall study quality (Figure 3).

### 3.2. Study Types

#### 3.2.1. Clinical Studies (Table S1)

##### Systematic Reviews

Amongst the identified clinical studies, we found nine systematic reviews.

Seven of these studies reported comparable outcomes with regard to flap survival, complication rates and adverse effects, regardless of the used vasoactive agent. No benefit was found for any particular antithrombotic drug and no consensus was found for a certain anticoagulation regime [1,13,14,15,16,17,18].

Two studies found higher risks for complications either with the combination of heparin and aspirin or with intravenous heparin in terms of bleeding. Low-molecular-weight dextran showed higher morbidity caused by pulmonary edema, nephrotoxicity or acute respiratory distress syndrome [19,20].

##### Metaanalyses

Two metaanalyses were identified, both showing rather equivalent flap survival rates when heparin or aspirin were used. Systemic complications may occur with the use of anticoagulants [21,22].

However, a high dose of dalteparin or heparin was associated with a greater flap loss rate than low dose use [21].

##### Prospective Randomized Clinical Trials (RCT)

Three RCT investigated systemic anticoagulants and topical irrigation substances. Low-molecular-weight dextran showed no improvement in free-flap survival but a significant degree of risk in causing systemic complications compared to aspirin [10].

Intraoperative intravenous milrinone did not show any benefit in flap survival nor in revision rates when compared to a saline infusion [23].

The intraluminal irrigation of flap vessels with heparin or low dose rhTFPI (0.05 mg/mL) may reduce the occurrence of postoperative hematoma and help to prevent flap failure [24].

##### Prospective Cohort Study or Case Series

A total of six prospective studies were identified. Four articles uniformly found similar outcomes in flap survival and complication rates when using heparin or aspirin.

One study additionally investigated additive leech therapy in single vs. multiple drug administration groups but found no overall differences [25].

Two studies demonstrated positive effects using either a continuous intravenous heparin infusion or intra-arterial irrigation during surgery in preventing thrombus formation [26,27].

##### Retrospective Cohort Study

The retrospective cohort study represents the most common article type, counting 21 studies.

Nine articles described no benefit from anticoagulative medication and similar complication rates [28,29,30,31,32,33,34,35]. The use of different medication, such as thrombolytics in flap failure or the use of Fogarty as a salvage procedure, made no difference [35]. Progressive tapering from intravenous anticoagulation in finger replantation even seemed to lower complication rates [29]. The use of VTE prophylaxis, however, in healthy patients or uncomplicated anastomosis is advocated [33,34,36].

Four studies reported similar clinical outcomes with heparin or dextran in terms of flap survival but found increased adverse effects, including postoperative bleeding or even flap failure, in high-risk patients [37,38,39,40].

Five studies reported benefits from additional heparin, ketorolac or surgical thrombectomy in terms of flap survival [6,41,42,43,44].

Kelly et al. reported non-significant results, observing that systemic anticoagulation and end-to-side anastomoses may be associated with lower rates of flap loss [45].

##### Others

Seven national or multinational multicenter surveys were found, describing a wide variety of applied anticoagulative strategies. One study acknowledged the heterogeneity and absence of standardized protocols [46]. Kremer et al. similarly pointed towards lacking advantages between diverse anticoagulants or in comparison to no anticoagulation at all [47].

According to Boyko et al., most US surgeons use anticoagulation or antiplatelet aggregation drugs postoperatively, with aspirin being the primary choice [48].

Xipoleas et al. reported about 84% of the participating surgeons using one or more anticoagulative agent and only 16.3% using no anticoagulation [4].

Rendenbach et al. found heparin use in 86%, mostly in therapeutic doses. Dextran was mostly started intraoperatively and continued for seven postoperative days [49].

Schmitz et al. tended towards low-molecular-weight heparin (LMWH) and balanced fluid supply. Aspirin was mostly used after replantation and less commonly following free flap surgery [50].

One single survey found the use dextran in 45%, occasionally in combination with aspirin or heparin [51].

Siegel et al. reported that aspirin, as an antithrombotic agent in microsurgery, exhibits optimal efficacy within the dosage range of 160 to 320 mg per day [52].

In a brief communication, Pederson et al. stated that prophylactic anticoagulation may be thoroughly considered when dealing with small caliber vessels, substantial size mismatch at the anastomosis site, utilization of vein grafts or compromised vessel quality only [53].

#### 3.2.2. Animal Studies (Table S2)

A total of 29 animal studies meeting the defined inclusion and exclusion criteria were identified. All studies (*n* = 29) were prospective cohort studies. Among these, 14 studies explicitly indicated that group allocation was randomized [54,55,56,57,58,59,60,61,62,63,64,65,66,67].

##### Prospective Cohort Studies/Randomized-Controlled Trials

(1) Systemic administration

The majority of studies reported favorable outcomes associated with the systemic administration of heparin in microsurgical procedures, particularly in terms of maintaining vessel patency and (flap) tissue survival: a randomized-controlled study performed in 54 rabbit ear replantations showed markedly enhanced arterial vessel patency and ear viability after one week by intravenous administration of 1000 IU of heparin after completion of the vessel anastomoses [65].

Femoral artery inversion grafts were also used to study the effect of either saline or heparin intravenous infusion over three days in the experimental rabbit model. Patency in the heparin group was 67%, compared to 19% in the control group infused with saline alone assessed on postoperative day seven [68].

Notably, even an intravenous bolus of 40 IU of heparin enhanced microvascular salvage after femoral artery thrombotic occlusion, extraction of the thrombus and vessel revascularization [60].

Compared to a control, arterial patency of groin/abdominal free flap vessels on postoperative day seven was significantly better after pentoxifylline and LMWH treatment. LMWH was administered subcutaneously and pentoxifylline via a gastric gavage tube four days pre- and six days postoperatively. No significant improvement was reported with the combination of both agents [61].

A randomized-controlled trial reported increased patency of microvascular anastomoses as well as increased tissue survival of epigastric free flaps at postoperative day seven with intraperitoneal administration of either unfractionated heparin (UFH, 100 IU/kg UFH in 1 mL of saline) or low-molecular-weight heparin (LMWH, 1 mg LMWH in 1 mL of saline with an average molecular weight of 4000 to 6000), compared to a control (1 mL of Ringer’s lactate), in a total of 66 rats. With regard to complications, hematomas occurred in only the UFH study cohort [63].

Only one randomized study from 1975 from Engrav et al. showed that a single dose of systemic 60 IU heparin had no benefit regarding thrombus prevention after partial amputation of hind legs in rat models followed by microvascular repair [57].

Nevertheless, numerous studies have conducted comparisons between systemic and local application of heparin. Across these studies, local application consistently demonstrated superior outcomes [69,70].

Li et al. investigated the timing of heparin treatment in microsurgical flap reconstruction. The authors were able to show that heparin protects from ischemia–reperfusion injury when it is administered either prior to or during flap ischemia. Histological examinations (dehydrogenase activity) of muscle flaps showed improved tissue viability with pre-ischemic heparin bolus administration or ex vivo washout with heparinized blood at different time intervals during flap ischemia, when compared to posttransplant heparinization or no heparinization [71].

In addition to heparin, some other agents showed positive effects on the microvasculature following systemic administration: a randomized-controlled study found beneficial effects of simvastatin on the microcirculation based on histological examination 48 h after free epigastric skin flap transplantation. Simvastatin counteracted prothrombotic conditions and induced vasodilation [54].

Another randomized-controlled study examined the effect of systemic intravenous tirofiban, combined with other anticoagulants or antiplatelet agents in 80 femoral vein anastomoses performed in a thrombogenic rat model. Combined therapy with a single dose of either aspirin/heparin or aspirin/heparin/tirofiban was associated with improved vessel patency 120 min after flap reperfusion and less thrombotic occlusion compared with controls [55].

(2) Irrigation/intraluminal and topical administration

Both intraluminal and topical administration/irrigation of micro-vessels demonstrated promising effects in animal studies.

Ramelli et al. have recently investigated the effect of intraluminal irrigation with unfractionated heparin (5000 IU/mL), compared to physiologic saline solution (0.9%), in 247 end-to-end anastomoses in rats performed by 21 surgeons. The primary endpoint was vascular 60 min thrombosis. The results showed a significant impact with a decrease in the thrombosis rate of 2.6 with heparin irrigation without increasing complications [72].

Accordingly, Cox et al. found that heparinized saline irrigation solution at a concentration of 100 IU/mL or greater significantly inhibited thrombus formation when using the same model of pedicle artery crush injury and microsurgical repair [56].

Korompilias et al. investigated different concentrations of enoxaparin as well as different application modes (topical irrigation versus systemic administration versus combination of both) in a vessel crush injury animal model. The results showed that high concentrations of topical enoxaparin (45 IU/mL, three times higher than doses recommended for clinical use adjusted by body weight) showed effective antithrombotic effects, while systemic administration alone did not prevent thrombus formation. A combination of systemic and local application was not associated with additional benefits regarding anastomotic patency [70].

Similar results were reported following transplantation of free musculocutaneous flaps to the superficial femoral artery when venous ischemia was induced by vessel clamps.

Six hours later, the clamps were removed, the venous anastomoses were revised and perfusion was restored. Then, heparin was infused either continuously locally via the inferior epigastric vein (200 IU heparin/1 cc of normal saline) or intravenously (5–6 IU/kg bodyweight/hour) and compared to a control without administration of heparin. Complete flap survival after seven days was observed in the local heparin group, while untreated animals and those receiving systemic heparin had flap loss rates of 60.8% and 62.1%, respectively [69].

Another study performed by Hudson et al. reported beneficial effects of local heparin infusion via catheters placed close to the site of the anastomosis on venous flow [73].

Several more studies reported beneficial effects of heparin as a microsurgical irrigant [62,74,75,76].

Addition of 20 IU/mL heparin to the irrigation solution (Ringer’s lactate) during rat groin flap revascularization failed to improve flap survival in one study only. It is suspected by the authors, however, that flap losses might have been caused by technical issues due to inexperience of the surgeon [64].

Wolfort et al. investigated the effect of regular pre- and postoperative dextran infusion compared to normal saline only. Inverted sleeve interposition grafts were placed at the arterial anastomosis, which triggered a thrombogenic response. Dextran significantly increased epigastric flap survival measured on postoperative day seven and was associated with higher vessel patency in electron microscopy [77].

In addition to heparin and dextran, irrigation with tirofiban, hirudin, urokinase, phentolamine, antithrombin and human tissue factor pathway inhibitor (TFPI) were suggested as effective vasoactive agents for intraluminal administration to prevent thrombus formation [58,62,66,67,74,75].

Lepore et al. infused selective drugs and drug mixtures intraarterially at the start of reperfusion of rabbit epigastric skin flaps. Compared with control, which had a 33% flap survival rate on postoperative day seven, vasodilators nitrendipine and prostacyclin and the thrombolytic agent urokinase showed improved outcomes. A combination of these agents was of particular benefit [78].

Another research group aimed their investigations at combinations of antithrombotic agents and radical scavengers to reduce reperfusion injury of flaps in a rabbit model. Significant improvements in flap take were obtained using intraarterial application of antithrombotic agents (heparin and urokinase) and radical scavengers (SOD and catalase) [79].

(3) Other routes of administration

Farina et al. demonstrated that moderate isovolemic hemodilution with 3% bovine albumin (30% hematocrit) is an effective way to reduce occlusion rates of venous anastomoses in rats 20 min and 48 h after the surgical procedure [80].

One study reported the use of heparin-coated vascular interposition tubes in venous anastomosis of experimental pigs. Ten days postoperatively, 90% of connectors demonstrated patency and adequate blood flow over the anastomosis. In the control group without heparin coating, only 45% of the same vascular tubes were open [81].

## 4. Discussion

The use of antithrombotic medication in free flap surgery and replantation is controversial. Drug regimens supporting blood rheology, counteracting platelet aggregation or venous thrombus formation, have been described. Additionally, the timing and mode of application can differ. This systematic literature review aims to further investigate the significance and safety of anticoagulants in microvascular free tissue transfer.

### 4.1. Clinical Studies

Among the clinical trials, only three RCTs were found, representing the highest research quality with a mean Coleman Score of 77. Two of these failed to show an advantage of low-molecular-weight dextran or intravenous milrinone to improve flap survival [10,23]. One study, however, showed a benefit of intraluminal vessel irrigation during the microvascular anastomosis with heparin or low-dose recombinant human tissue factor pathway inhibitor. They found a diminished occurrence of postoperative bleeding and decreased flap failure. Thereby, Khouri et al. remain the only published RCT to date reporting a positive outcome regarding a technique to improve flap survival. Interestingly, no other clinical trial was conducted to confirm and validate these findings [24].

Despite representing the best research quality, none of these studies compared the most commonly investigated drugs and application methods in the literature. These are subcutaneous heparin/LMWH and intravenous or oral aspirin.

Research on these drugs is mostly provided by only the prospective and retrospective studies without adequate control groups. The second-most valuable article type is the prospective cohort study, which was represented by only six items. The majority of these articles found similar results in terms of flap survival and complication rates when using heparin or aspirin [7,25,82,83].

Notably, one of the prospective studies reported positive results using an intravenous infusion via a teflon catheter inserted into the arterial lumen at the site of the anastomosis. This study was rated, however, with a low Coleman score of only 32.

More uniform results were demonstrated by the high number of retrospective cohort studies, counting 21 items. Nine described no benefit from antithrombotic or antiplatelet aggregation drugs and equal complication rates [28,29,30,31,32,33,34,35]. Four studies reported comparable clinical outcomes with heparin or dextran. However, they found increased adverse effects, including postoperative bleeding or even flap failure in high-risk patients [37,38,39,40]. Only five studies found benefits from heparin, ketorolac or surgical thrombectomy during salvage in terms of flap survival [6,41,42,43,44]. One study tended towards positive effects; however, the results were not statistically significant [45].

Among the review articles, we found two metaanalyses and nine systematic reviews, both depicting a high research quality. The results of the two metaanalyses are in line with the majority of the pro- and retrospective studies, showing similar flap survival rates when heparin or aspirin was used. Systemic complications were reported with the use of anticoagulants [21,22]. However, a procedure-related complication was noted for high-dose dalteparin or heparin in terms of a greater flap loss rate compared to low dose use [21].

Likewise, seven systematic reviews reported similar outcomes with regard to flap survival, complication rates and adverse effects. No advantage was found for any particular anticoagulation regime or specific drug [1,13,14,15,16,17,18]. Two studies found higher odds for complications either with the combination of heparin and aspirin or intravenous heparin in terms of postoperative hematoma. Low-molecular-weight dextran showed a higher morbidity caused by its oncotic properties in terms of pulmonary edema, nephrotoxicity or acute respiratory distress syndrome [19,20]. Notably, dextran was reported to be used intraoperatively and continued for seven days throughout the operation [49].

An association of dextran and postoperative complications without a clear benefit on flap survival was reported by several studies; therefore, its administration is currently not recommended [5,84,85,86].

The most uniform findings in our investigation were the results of the multicenter surveys with no matching conclusions. In the United States, most surgeons use anticoagulation post-operatively, with aspirin being the primary drug of choice [48]. Contrarily, Rendenbach et al. found heparin use in 86%, mostly in therapeutic doses [49]. Europeans instead use LMWH and balanced fluid supply. Aspirin was mostly used after limb replantation, not after free flap surgery [50].

We support the results of Schmitz et al. as, in our center, free flaps below the diaphragm receive half-therapeutic LMWH plus a single shot of 2000 IE intravenous heparin prior to the microvascular anastomosis, while flaps above the diaphragm receive LMWH as deep venous thrombosis prophylaxis only.

### 4.2. Animal Studies

While clinical data regarding potentially beneficial anticoagulation regimens in microsurgery remained rather inconclusive, the reviewed animal studies provided some valuable insights to optimize microvascular outcomes using vasoactive substances.

A diverse range of animal studies investigated the effects of heparin and other anticoagulants, including systemic administration, intraluminal irrigation and topical application.

The majority of studies observed favorable outcomes in terms of enhanced vessel patency and tissue survival following systemic administration of heparin [60,61,63,65,68]. However, there was a wide range of study designs, including variations in dosage and timing of administration, highlighting the need for standardized protocols in future investigations.

Favorable microvascular outcomes were consistently reported, in particular with local or intraluminal application of heparin in animal models [56,69,70,72]. In addition to its systemic anticoagulative action, heparin is believed to elicit protective vascular effects directly at the vessel endothelium. This might be one approach to explain the potent action of a targeted heparin administration [69].

While patient populations in clinical trials are inherently more heterogenous compared to laboratory animal cohorts, the concentration and duration of local heparin application that proved effective in animal studies might not be easily translatable to human subjects for safety reasons. Korompilias et al. investigated a range of concentrations of enoxaparin in a vessel crush injury animal model. Relatively high concentrations of enoxaparin, exceeding the doses recommended for clinical use when adjusted for body weight, had protective antithrombotic effects in several animal studies [56,70,72]. It might be challenging, however, to achieve therapeutic heparin levels at the site of vessel anastomosis without risking adverse systemic effects in human studies.

Further to route and dosage, timing of heparinization might be another point of interest. The histological examinations performed by Li et al. revealed improved flap tissue viability with a pre-ischemic heparin bolus or flap washout with heparinized blood during flap ischemia rather than posttransplant heparinization [71]. To date, there is no conclusive evidence to support the preoperative start of heparin prophylaxis to prevent deep venous thrombosis and pulmonary embolism in surgical patients [87]. Accordingly, thrombosis prevention using subcutaneous enoxaparin is frequently started postoperatively in plastic surgery procedures. Taking into account the insights of the presented animal study by Li et al., pre- and intraoperative heparinization might be considered in free flap cohorts to prevent microvascular failure [71]. However, further investigations under careful observation of potential bleeding complications in a controlled study setting are necessary prior to any clinical translations.

Despite the valuable insights provided by the reviewed studies, certain limitations should be acknowledged.

Firstly, due to restricted access at our university, we were unable to explore databases beyond PubMed.

Secondly, the variability in patient cohorts, surgical techniques, vasoactive agents, dosage and timing of administration, as well as outcome measures, challenge a generalized conclusion. Future research should first focus on a standardization of the methodologies.

## 5. Conclusions

In conclusion, this systematic review demonstrates the lack of a clear consensus in the current literature on anticoagulation in microsurgery. The reviewed studies were heterogenous in quality, reflected by a mean overall Coleman score of nearly 50% of achievable points. Clinical studies presented diverse perioperative regimens, particularly centered around aspirin, heparin and dextran. The metaanalyses highlighted the need for cautious dosing of heparin and LMWH. Dextran is not generally recommended. Animal studies suggested potential benefits with systemic and with topical heparin application in particular.

The overall uncertainty coupled with the low Coleman score emphasize the necessity for more high-powered, high-quality randomized clinical trials with adequate control groups. Addressing these gaps is essential to establish evidence-based practices and guide optimal clinical decision-making in microsurgical procedures.

## Figures and Tables

**Table 1:** Study eligibility criteria.

Category	Inclusion Criteria	Exclusion Criteria
Characteristics	🗸 Sample ≥ 10 🗸 Clinical studies 🗸 Animal studies 🗸 Free-flap transfers and replantation in any body region 🗸 Anticoagulants, antiplatelet drugs, drugs influencing rheological behavior	🗴 States of hypocoagulability and hypercoagulability that were not drug-induced 🗴 Extracorporeal perfusion
Intervention	🗸 Intra- and postoperative anticoagulation therapy	🗴 No anticoagulation treatment
Outcomes	🗸 Thromboembolic complications, flap loss, bleeding complications, revision surgery	
Study design	🗸 Metanalysis 🗸 Systematic review article 🗸 Randomized controlled clinical trial 🗸 Prospective or retrospective controlled clinical trial 🗸 Case–control study	🗴 Case report 🗴 No control study 🗴 Non-systematic literature reviews 🗴 Interviews 🗴 Commentaries 🗴 Conference abstracts 🗴 Replies to the editor/author

## Data Availability

All data originally created by the authors themselves during the preparation of this systematic literature review are reported in the presented manuscript.

## Supplementary Materials

The following supporting information can be downloaded at: https://www.mdpi.com/article/10.3390/life14010082/s1, Table S1: Clinical Studies; Table S2: Animal Studies.
