# Supplementary material for: Evidence-Based Approaches to Anticoagulation in Reconstructive Microsurgery—A Systematic Literature Review
Source: Life (Basel). 2024 Jan 3;14(1):82. doi: 10.3390/life14010082 (PMC10817551; doi:10.3390/life14010082)
Supplement: Supplementary file 1 [file life-14-00082-s001.zip › life-2763222-supplementary/Table S1 Clinical Studies .pdf]

1st Author	Year of Publica-tion	Recipient Site	Number of anticoagu-lants used	Medication/method of administration	Time of application	Number of participants/ Flaps	Age, y	Sex (male/ female)	Flap Loss-Total (*) %	Revision surgery (*) %	Hemato-ma (*) %	Overall Compli-cation Rate (*) %	CMS	Summary
Metaanalysis														
Pan et al.	2014												73	No significant difference in flap loss rate between heparin and ASA; high dose dalteparin or heparin associated with a greater flap loss rate than low dose.
Swartz et al.	2015												77	Studied anticoagulants do not improve free flap survival, in addition some anticoagulative drugs may cause systemic complications.
Systematic Review														
Abraham et al.	2008	Head, neck											71	Subcutaneous heparin and ASA are commonly combined, but the combination also increases the rate of complications. ASA as single-agent does not increase flap survival. Recent studies show higher benefit, safety, and decreased risk of bleeding with LMWH alone.
Cevik et al.	2021												81	LMWH and Heparin are

														equivalent as prophylaxis. Heparin administered three times daily should be considered.
Couteau et al.	2021												72	Intraoperative systemic heparin does not improve flap survival. Ex vivo administration of intraoperative irrigation with heparinized serum during cold ischemia time, improves free flap survival.
Lin et al.	2020	Digital replanta-tion											71	No benefit LMWH vs UFH concerning success rates of replantation. Similar rates of complications and adverse effects.<br>No difference in anastomosis sufficiency between LMWH and no LMWH.
Liu et al.	2017	Head, neck											63	No significant difference between postop. anticoagulation therapy and flap loss rate/thromboembolic event. Postoperative anticoagulation therapy may increase the

														risk of hematoma
Prsic et al.	2015												51	Anticoagulation with ASA, heparin and dextrane described in literature. No consensus about the ideal regimen. Use of statin perioperatively could be beneficial.
Sigaux et al.	2017												45	No significant difference in flap outcome or complication rate between the common anticoagulatives. Important: Monitoring of blood results (HIT) and individual regimen
Spiegel et al.	2007												50	LMW-dextran: higher morbidity caused by pulmonary edema, nephrotoxicity, acute respiratory distress syndrome, severe anaphylactic reaction. intravenous heparin: increased risk of hematoma, heparin subcutaneous same risk as no anticoag.

														ASA: no increased risk for complications compared with patients receiving no anticoagulant.
Trull et al.	2021												44	Mostly used anticoagulation: ASA alone or in combination with UFH/LMWH. No clear consenus.
Randomized Controlled Trial														
Disa et al.	2013	Head, neck	Total: 3 (1 each group)	Group 1: low-molecular-weight dextran<br>Group 2: low-molecular-weight dextran<br>Group 3: ASA	48h postoperatively<br>120h postoperatively<br>5 days postoperatively	Group 1: 35<br>Group 2: 32<br>Group 3: 27	58<br>59<br>57	54/46<br>72/28<br>63/37	0<br>0<br>0	0<br>2 (6.2%)<br>0	N.a.<br>N.a.<br>N.a.	7 (20%)<br>2 (6.2%)<br>2 (7.4%)	83	
Jones et al.	2007	mixed	Total: 2	Group 1: 200µg/ml milrinone bolus<br>Group 2: saline infusion	intraoperatively	Group 1: 43<br>Group 2: 45	51 (18-80)<br>51 (20-78)	17/26<br>17/28	(1) 2%<br>(1) 2%	(10) 23%<br>(7) 16%	(5) 11%<br>(4) 9%	N.a.	73	
Khouri et al.	2001	mixed	Total: 2	Group 1: 0.05mg/ml rhIFPI<br>Group 2: 0.15mg/ml rhIFPI<br>Group 3: 100hU/ml heparin	intraluminal irrigation	Group 1: 216<br>Group 2: 204<br>Group 3: 202	47 (17-89)<br>45 (18-88)<br>46 (18-84)	61/39<br>60/40<br>59/41	(4) 1.9%<br>(13) 6.4%<br>(10) 5.0%	10.4 %<br>9.3%<br>7.9%	(7) 3.2%<br>(17) 7.8%<br>(18) 8.9%	N.a.	76	
Prospective Cohort Study														
Ashjian et al.	2007	mixed	Group 1: 1<br>Group 2: 1	Group 1: 325mg of ASA<br>Group 2: 5000 units LMWH	daily for 5 days<br>daily until ambulating	245/260<br>225/245	57 (10-102)<br>54 (3-89)	101/144<br>102/123	(1) 0.4%<br>(2) 0.8%	N.a.<br>N.a.	(6) 2.3%<br>(5) 2%	37.7<br>44	75	
Bashir et al.	2014	mixed	Total: 1	Group 1: 5000 units of heparin IV, no heparin given postoperatively<br>Group 2: 5000 units of heparin IV per operatively, 1000 units of heparin IV per hour were given postoperatively	intraoperatively<br>Intra- and post-operatively	Group 1: 16<br>Group 2: 15	29.93<br>35.53	2,7:1 ratio<br>4:1 ratio	(3) 19%<br>(4) 23%	1<br>2	(2) 12.5%<br>(5) 33.3%	N.a.<br>N.a.	72	
Fukui et al.	1987	Upper extremity	Total: 1	IV infusion via Teflon catheter inserted into proximal main artery of the anastomosis, daily dose of 80ml comprising 240.000U of urokinase, 40µg of prostaglandin E, 10.000U (maximum) of heparin and low molecular weight dextran	continuous infusion for the 10 consecutive days	15	N.a.	N.a.	0	N.a.	N.a.	N.a.	32	
Furnas et al.	1992	Head, neck	Total 3:		Total: 55	N.a.		N.a.	N.a.	N.a.	N.a.	N.a.	42	

				Group 1: 325mg ASA, dextran<br>Group 2: 325mg ASA, heparin<br>Group 3: 325mg ASA, dextran replaced by heparin<br>Group 4: 325mg ASA, dextran, leeches<br>Group 5: 325mg ASA, extran, heparin<br>Group 6: 325mg ASA, heparin, leeches<br>Group 7: 325mg ASA, heparin, dextra, leeches	Group 1: 28<br>Group 2: 8<br>Group 3: 4<br>Group 4: 5<br>Group 5: 5<br>Group 6: 2<br>Group 7: 3		3%<br>25%<br>30%<br>27%<br>25%<br>40%<br>0%							
Lighthall et al.	2011	Upper and lower extremity	Group 1: 1<br>Group 2: 1<br>Group 3: 1<br>Group 4: 2<br>Group 5: 0	Group 1: ASA<br>Group 2: heparin<br>Group 3: heparin drip<br>Group 4: heparin and ASA<br>Group 5: none	N.a.	Group 1: 142<br>Group 2: 25<br>Group 3: 15<br>Group 4: 23<br>Group 5: 184	64.3	193/197	(7) 4.9%<br>(2) 8%<br>(5) 31.3%<br>(1) 4.4%<br>(10) 5.4%	N.a.	(10) 7.04%<br>(6) 24%<br>(5) 33.33%<br>0<br>(7) 3.80%	(57) 40%<br>(12) 48%<br>(21) 100%<br>(15) 65%<br>(45) 24%	48	
Saito et al.	2009	Lower extremity	Total: 1	2000IU of heparin and 4lg of PGE1	continuous through catheter for 7 days	11	46.8	5/6	0%	0%	9.09%	36.36%	60	
Başaran et al.	2021	Head, neck	Group 1: 2<br>Group 2: 1	Group 1: enoxaparin 2x4000IU, ASA 1x100mg<br>Group 2: enoxaparin 6000IU	N.a.<br>N.a.	Total: 124<br>Group 1: 63<br>Group 2: 61	45.9 (14–72)	67/57<br>N.a.<br>N.a.	(3) 0.05%<br>(0) 0%	(22) 17.7%<br>(15) 24%<br>(6) 9.8%	(18) 14.5%<br>(12) 19%<br>(6) 9.8%	N.a.	63	
Bassiri-Tehrani et al.	2018	Breast	Total: 2	Group 1: preoperative chemoprophylaxis, 40mg enoxaparin SC<br>and additionally 81mg ASA<br>Group 2: postoperative chemoprophylaxis, enoxaparin SC<br>and additionally 81mg ASA	intraoperatively, until ambulating<br>early postoperatively<br>postoperatively, until ambulating<br>early postoperatively	Group 1: 105/166<br>7 of Group 1<br>Group 2: 91/145<br>29 of Group 2	51.4<br>48.1	N.a.	N.a.	N.a.	(4) 4.4%<br>(1) 1%	(4) 3.8%<br>(6) 6.7%	53	
Efanov et al.	2018	Upper extremity	Total: 2	Group 1: 80mg ASA and heparin IV<br>Group 2: 80mg ASA and heparin IV<br>Group 3: 80mg ASA and heparin IV	ASA daily,<br>heparin for ≤ 7 days<br>heparin for 7-14 days<br>heparin for ≥ 15 days	Total: 108/163<br>36<br>67<br>5	48 (16-76)	96/12	16 (44.4%)<br>24 (35.8%)<br>3 (60.0%)	N.a.<br>N.a.<br>N.a.	N.a.<br>N.a.<br>N.a.	N.a.<br>N.a.<br>N.a.	65	
Eley et al.	2013	Head, neck	Total: 2	Group 1: 2500IU Dalteparin, 75mg ASA<br>Group 2: 2500IU Dalteparin<br>Group 3: 5000IU Dalteparin, 75mg ASA<br>Group 4: 5000IU Dalteparin<br>Group 5: 5000IU Dalteparin, 75mg ASA<br>Group 6: 5000IU Dalteparin<br>Group 7: 7500IU Dalteparin, 75mg ASA<br>Group 8: 7500IU Dalteparin	once a day<br>once a day<br>once a day<br>once a day<br>twice a day<br>twice a day<br>once a day<br>once a day	Total: 173<br>5<br>2<br>78<br>29<br>22<br>15<br>15<br>5	N.a.	101/72	N.a.	0<br>0<br>(4) 5.1%<br>(1) 3.4%<br>(1) 4.5%<br>0<br>(2) 13.3%<br>0	0<br>0<br>(3) 3.8%<br>(1) 3.4%<br>(2) 9.0%<br>0<br>0<br>0	N.a.	76	
Enajat et al.	2014	Breast	Total: 2	Group 1: 0.6ml nadroparine and 40mg ASA<br>Group 2: 0.6ml nadroparine	nadroparine until discharge, ASA daily for 6 weeks postoperatively	Total: 430/592<br>Group 1: 261<br>Group 2: 169	47.5<br>47.8	0/261<br>0/169	2.8%<br>2.5%	N.a.	9.2%<br>4.7%	28.0<br>32.3	66	

Filipan et al.	2020	Head, neck	Total: 2	Group 1: dextran 40, 500mL/24 hours, dalteparin 5000IU daily<br>Group 2: dalteparin 5000IU daily	dextran 40 for 1-6 days, dalteparin for an average of 7 days	Total: 168/176<br>Group 1: 97<br>Group 2: 79	59.9<br>58.8<br>61.3	118/58<br>69/28<br>49/30	16<br>10<br>6	27<br>(13) 13.4%<br>(14) 17.7%	(11) 11.3%<br>(3) 3.8%	(13) 13.4%<br>(10) 12.7%	66	
Jayaorasad et al.	2013	Head, neck	Total: 1	Group 1: 50g dextran 40, LMWH<br>Group 2: LMWH only	dextran 40 for daily for 5 days postoperatively, LMWH 0.2cc SC daily for 5 days or until ambulation	Total: 169<br>Group 1: 86<br>Group 2: 82	55.6<br>(18-85)	62/24<br>57/25	(3) 3.5%<br>(2) 2.3%	(26) 15.4%<br>(12) 14%<br>(14) 16.3%	(5) 5.8%<br>(2) 2.4%	N.a.	74	
Keith et al.	2013	Breast	Total: 1	Group 1: only postoperative enoxaparin 1x40mg or 2x30mg daily<br>Group 2: preoperative and postoperative 30mg enoxaparin SC	Group 1: postoperatively daily until ambulation<br>Group 2: within 1 hour before incision, postoperatively according to group 1	Group 1: 58<br>Group 2: 89	N.a.	N.a.	N.a.	(1) 1.7%<br>(3) 3.4%	(2) 3.4%<br>(5) 5.6%	N.a.	71	
Kelly et al.	2004	mixed	Total: 3	Group 1: LMWH<br>Group 2: no anticoagulation<br>Group 3: dextran<br>Group 4: systemic heparin	Group 3: 3-5 days<br>Group 4: 3-5 days	Group 1: 39<br>Group 2: 10<br>Group 3: 6<br>Group 4: 4	N.a.	N.a.	(5) 12%<br>(4) 0.4% *<br>0<br>0<br>*(total and partial flap loss)	N.a.	N.a.	N.a.	55	
Labovsky et al.	1991	Lower extremity	Group 1: 3<br><br>Group 2: 3	Group 1: 625mg ASA supp<br>5000IE Heparin IV<br>Dextran IV<br>625mg ASA<br><br>Group 2: 625mg ASA supp<br>Heparin IV venous catheter in anastomosis (5IE/cc, 50cc/hr)<br>Dextran IV<br>625mg ASA	preoperatively<br>intraoperatively<br>3 days postoperatively<br>postoperatively<br><br>preoperatively<br>3 days intraoperatively<br><br>3 days postoperatively<br>postoperatively	41<br><br>36		0<br><br>(1) 6.67 %		(8) 61.54%<br><br>(1) 6.67%	N.a.	N.a.	53	
Lee et al.	2012	Lower extremity	Group 1: 2<br><br>Group 2: 1	Group 1: prostaglandin E1 10µg,<br>Keterolac 30mg IV 1-1-1<br>Group 2: prostaglandin E1 10µg	7 days postoperatively<br>2 days postoperatively<br>7 days postoperatively	Group 1: 80<br><br>Group 2: 48	45.5<br><br>48	54/26<br><br>25/23	(1) 1.3%<br><br>(1) 2.1%	(4) 5%<br><br>(8) 16.7%	(1) 1.3%<br><br>(3) 6.3%	(7) 8%<br><br>(12) 25%	68	
Luthringer et al.	2022	Head, neck	Group 1: 1<br><br>Group 2: 1-2	Group 1: 15mg ketorolac<br><br>Group 2: ASA 100 (some)<br>LMWH	Group 1: intraoperatively (time of anastomosis), every 6 hours thereafter for max. 5 days<br>Group 2: daily	Group 1: 18<br><br>Group 2: 6	63.2<br><br>59.7	12/6<br><br>5/1	0<br><br>0	0<br><br>0	0<br><br>0	N.a.	55	
Mirzabeigi et al.	2012	mixed	Total: 1	LMWH 5000IE<br>some unclear	intraoperatively	47	51.2	N.a.	51%	100%	N.a.	N.a.	41	
Numajiri et al.	2016	Head, neck	Group 1: 1<br><br>Group 2: 0	Group 1: continuous IV UFH (5000–10 000 units/d)<br>Group 2: none/control	7d postoperatively	Group 1: 121<br>Group 2: 62	60.6<br>61.9	92/29<br>57/5	3.3%<br>1.6%	4.1%<br>1.6%	8%<br>0%	N.a.	79	
Okochi et al.	2019	Lower extremity	Total: 1	Group 1: heparin 5.000-10.000IU/d (arterial catheter)<br>Group 2: 50% of the initial dose<br>Group 3: 25% of the initial dose of heparin	until 2.POD<br><br>until 4.POD<br>until 6.POD	15	55.1	10/5	0%	N.a.	33.33%	N.a.	53	

Pomerance et al.	1994	Digital replanta-tion		mostly LMW- dextran 500cc/day<br>and ASA	until 3. POD	151 digits	32.7	93/13	12%	13%	N.a.	N.a.	29	
Pugh et al.	1996	Lower extremity	Total: 3	Group 1: ASA<br>Group 2: dextran<br>Group 3: heparin, ASA<br>Group 4: heparin, dextran<br>Group 5: heparin, dextran, ASA	intraoperatively	Total: 15/16<br>Group 1: 5<br>Group 2: 4<br>Group 3: 1<br>Group 4: 4<br>Group 5: 1	33.8	13/2	6.25%<br>0%<br>25%<br>0%<br>0%<br>0%	18.75%<br>0%<br>50%<br>100%<br>25%<br>100%	0%<br>0%<br>100%<br>25%<br>100%	N.a.	36	
Riva et al.	2012	Head, neck	Group 1: 0<br>Group 2: 1<br>Group 3: 0	Group 1: PGE1<br>Group 2: dextran 40<br>Group 3: none	Intra- and postoperatively	total 1351<br>Group 1: 232<br>Group 2: 283<br>Group 3: 836	51.7<br>51.5<br>52.1	N.a.	6%<br>6%<br>5%	15.9%<br>11.0%<br>12.0 %	4.7%<br>1.8%<br>2.2%	N.a.	63	
Senchenkov et al.	2015	mixed	Total: 2-4	5000IE heparin<br>3000-5000IE heparin<br>ASA oral/rectal, Heparin weight based<br>tPA, heparin weight based and dextran	preoperatively<br>before anastomosis<br>postoperatively<br>in case of thrombosis	255/395	51.0	47/208	0.5%	9.4%	27%	N.a.	49	
Tamai et al.	1977	Digital replanta-tion	Group 1: 3<br><br><br><br>Group 2: 3	Group 1: heparin 20.000IU/day,<br>LMW dextran 500-1000IU/day,<br>urokinase 30.000-50.000IU/day,<br>with intraoperative irrigation<br><br>Group 2: heparin 20.000IU/day,<br>LMW dextran 500-1000IU/day,<br>urokinase 30.000-50.000IU/day,<br>without intraoperative irrigation	until 5-10. POD<br>until 3-5. POD<br>until 7-10. POD<br><br>until 5-10. POD<br>until 3-5. POD<br>until 7-10. POD	62<br><br><br><br>80	45.5<br><br><br><br>45.5	N.a.	17.74%<br><br><br><br>6.25%	N.a.	N.a.	N.a.	53	
Yii et al.	2000	mixed	Total: 2	Urokinase and/ or recombinant tissue–plasminogen activator intraluminal	Group 1: salvage group<br>Group 2: flap failure group	1733 flaps, 55 reexplored, 41 of them with thrombosis<br>Group 1: 28<br>Group 2: 13	49<br>50	8/20<br>4/8	0%<br>100%	(28) 100%<br>(13) 100%	N.a.	N.a.	39	
Others														
Boyko et al.	2019	mixed											24	
Davies	1982												36	
Kremer et al.	2016												37	
Pederson	2008	Lower extremity											25	
Rendenbach et al.	2017												26	

Ridha et al.	2005												28	
Schmitz et al.	2011												29	
Siegel	1991												37	
Xipoleas et al.	2007												28	
