# Supplementary material for: Evidence-Based Approaches to Anticoagulation in Reconstructive Microsurgery—A Systematic Literature Review
Source: Life (Basel). 2024 Jan 3;14(1):82. doi: 10.3390/life14010082 (PMC10817551; doi:10.3390/life14010082)
Supplement: Supplementary file 1 [file life-14-00082-s001.zip › life-2763222-supplementary/Table S2 Animal Studies.pdf]

1st Author	Year of Publi-cation	Recipient Site	Number of anticoagulants used	Medication/method of administration	Time of application	Number of participants/ Flaps	Flap Loss-Total (*) %	Hemato-ma (*) %	Overall Compli-cation Rate (*) %
Prospective Cohort Study									
Abdelfattah et al.	2020	Chest	Total: 1	Group 1: NaCl<br>Group 2: Simvastatin<br>Group 3: Simvastatin and ASA	daily for 7 days<br>daily for 7 days<br>daily for 7 days	Group 1: 12/24<br>Group 2: 12/24<br>Group 3: 12/24	(6) 25%<br>(3) 12.5%<br>(2) 8.3%	0<br>0<br>1	N.a.
Chung et al.	2016	Lower extremity	Total: 4	Group 1: ASA and heparin<br>Group 2: ASA, heparin and tirofiban<br>Group 3: tirofiban<br>Control group 4: isotonic saline	N.a.	Group 1: 20<br>Group 2: 20<br>Group 3: 20<br>Group 4: 20	N.a.	N.a.	30%<br>0%<br>70%<br>80%
Cox et al.	1992	Lower extremity	Total: 1	Control group 1: normal saline<br>Group 2: heparinised saline (10U/ml)<br>Group 3: heparinised saline (100U/ml)<br>Group 4: heparinised saline (250U/ml)<br>Group 5: heparinised saline (500U/ml)	intraoperative irrigation	Group 1: 8<br>Group 2: 8<br>Group 3: 8<br>Group 4: 8<br>Group 5: 8	N.a.	N.a.	(8) 100%<br>(5) 62.5%<br>(1) 12.5%<br>(0)<br>(1) 12.5%
Engrav et al.	1975	Lower extremity	Total: 2	Group 1: control group<br>Group 2: 60IU heparin systemically<br>Group 3: magnesium dripped on the anastomosis<br>Group 4: 60IU heparin systemically, magnesium dripped on the anastomosis	Group 2: after opening the venous anastomosis<br>Group 3: 20min with the artery being open inter-mittently and the vein continously<br>Group 4: according to group 2+3	Group 1: 12<br>Group 2: 11<br>Group 3: 10<br>Group 4: 11	(3) 25%<br>(1) 9%<br>(4) 40%<br>(2) 18%	N.a.	N.a.
Ezzat et al.	2010	Lower extremity	Total: 1	Group 1: rhTPFI (recombinant human tissue factor pathway inhibitor)<br>Group 2: control group	Group 1: irrigation with rhTPFI (20µg/mL)<br>Group 2: irrigation with saline	Group 1: 10<br>Group 2: 9	0<br>(4) 44%	0<br>(1) 11%	N.a.
Farina et al.	2006	Lower extremity	Total: 2	Group 1: control group<br>Group 2: hemodiluted until hematocrit of +/- 30%<br>Group 3: dextran<br>Group 4: enoxaparin	Group 3: 3 doses in an interval of 20min, 5min before surgery<br>Group 4: 0.5mg/kg once a day	Group 1: 10<br>Group 2: 10<br>Group 3: 10<br>Group 4: 10	N.a.	N.a.	100%<br>50%<br>90%<br>100%
Greenberg et al.	1988	Lower extremity	Total: 1	Group 1: saline (control group)<br>Group 2: heparin	Group 2: 500µl bolus, 45µl per hour after graft was completed	Group 1: 30<br>Group 2: 30	(30) 100%<br>(35) 100%	0<br>(1) 3%	N.a.
Greenberg et al.	1991	Lower extremity	Total: 1	Group 1: saline (control group)<br>Group 2: heparin intravenous<br>Group 3: heparin intraarterial	Group 2: 500µl bolus, 45 units per hour<br>Group 3: 500µl bolus, 25 units per hour	Group 1: 12<br>Group 2: 12<br>Group 3: 11	(9) 92%<br>(6) 50%<br>(2) 18%	0<br>(1) 8%<br>(1) 9%	N.a.

Hudson et al.	2000	Lower extremity	Total: 1	Group 1: saline (control group)<br>Group 2: 5000IU heparin	via catheter proximal to the anastomosis	Group 1: 10<br>Group 2: 10	(10) 100%<br>(9) 90%	N.a.	N.a.
Kirschner et al.	1998	Lower extremity	Total: 1	Group 1: control group<br>Group 2: low dose heparin systemically following secondary venous ischemia<br>Group 3: low dose heparin locally into postischemic flap	Group 2 and 3:<br>5-6U/kg/h for 7 days	Group 1: 9<br>Group 2: 9<br>Group 2: 9	60.8%<br>62.1%<br>0	0<br>0<br>0	N.a.
Korompilias et al.	1996	Lower extremity	Total: 1	Group 1: systemic heparin 2x45IU<br>Group 2: topical heparin 15IU/ml<br>Group 3: topical heparin 45IU/ml<br>Group 4: systemic 2x15IU/ml and topical 15IU/ml heparin<br>Group 5: systemically 2x45IU/ml and topical 45IU/ml heparin	Group 1: systemically<br>Group 2: topical irrigation<br>Group 3: topical irrigation<br>Group 4: systemically and topically<br>Group 5: systemically and topically	Group 1: 20<br>Group 2: 20<br>Group 3: 20<br>Group 4: 20<br>Group 5: 20	(19) 95%<br>(13) 65%<br>(8) 40%<br>(10) 37.5%<br>(4 rats died)<br>(5) 25%	N.a.	N.a.
Lan et al.	1992	Lower extremity	Group 1: 1<br>Group 2: 1<br>Group 3: 1	Group 1: saline<br>Group 2: iloprost<br>Group 3: heparin	postoperatively	Group 1: 48<br>Group 2: 48<br>Group 3: 48	N.a.	N.a.	N.a.
Lepore et al.	1994	Lower extremity	Total: 6	Group 1: Prostacyclin 200mg/kg<br>Group 2: Nitrendipine 0.2mg/kg<br>Group 3: Desferrioxamine 20mg/kg<br>Group 4: Urokinase 3000U/kg<br>Group 5: Streptokinase 3000U/kg<br>Group 6: Heparin 200U/kg<br>Group 7: Macrodex 80mg/kg<br>Group 8: ATP-MgCI, 20rng/kg<br>Group 9: Mixture PGI2 200ng/kg, Desferrioxamine 20mg/kg, ATP-MgCl 10mg/kg, Macrodex 1ml, Heparin 200 units/kg, Nitrendipine, 0.2mg/kg	intraoperatively		Group 1: 65%*<br>Group 2: 62%*<br>Group 4: 65%*<br>Group 9: 87%*<br>*survival	N.a.	N.a.
Li et al.	1995	A./V. femoralis	Total: 1	Group 1: heparin (150 units/kg)<br>Group 5: heparin (150 units/kg)<br>Group 2-4: no heparin/ex vivo perfusion<br>Group 6: none	Group 1: 10min prior to raising the flap<br>Group 5: after the ischemic period, immediately<br>Group 2-4: prior to recirculation of the flap via cannula with heparinised blood	Group 1: 6<br>Group 5: 6<br>Group 2-4: 3x6<br>Group 6: 6	100%<br>100%<br>100%<br>66.6%	N.a.	N.a.<br>33.3%
Maeda et al.	1990	A./V. epigastrica<br>A./V. femoralis	Group 1a: 0<br>Group 2a: 2<br>Group 3a: 0<br>Group 4a: 0<br>Group 1b: 0<br>Group 2b: 2	Group 1: none<br>Group 2: heparin, urokinase<br>Group 3: Ringer Lactat<br>Group 4: SOD, Catalase, Ringer Lactat<br>Group 1b: none<br>Group 2b: heparin, urokinase	30min before reperfusion after 10h<br>30min before reperfusion after 10h<br>30min before reperfusion after 10h<br>30min before reperfusion after 10h<br>30min before reperfusion after 12h<br>30min before reperfusion after 12h	12<br>10<br>10<br>Group 4a: 10<br>Group 1b:10<br>10	46%<br>0%<br>32%<br>47%<br>87%<br>73%	N.a.	N.a.

			Group 3b: 0<br>Group 4b: 0<br>Group 5b: 2	Group 3b: Ringer Lactat<br>Group 4b: SOD, Catalase, Ringer Lactat<br>Group 5b: heparin, urokinase, SOD, Catalase, Ringer Lactat	30min before reperfusion after 12h<br>30min before reperfusion after 12h<br>30min before reperfusion after 12h	10<br>10<br>10	84%<br>64%<br>5%		
Mayer et al.	1990	V. axillaris<br>V. jugularis int.<br>V. subscapularis	Group 1: 1<br>Group 2: 0	heparin coated	on surface of interposition connectors	Group 1: 20 anastomosis in 6 pigs<br>Group 2: 30 anastomosis in 7 pigs			
Murthy et al.	2003	A./V. epigastrica inf.	Group 1: 0<br>Group 2: 1<br>Group 3: 1<br>Group 4: 2	control group<br>pentoxifylline<br>LMWH<br>combined therapy (pentoxifylline and LMWH)	Group 2: 24mg/kg/day<br>Group 3: 1.5mg/kg/day	Group 1: 15<br>Group 2: 15<br>Group 3: 15<br>Group 4: 15	66.67%<br>26.67%<br>26.67%<br>20%	N.a.	N.a.
Narayanan et al.	1991	A.femoralis<br>V.femoralis	Group 1: 0<br>Group 2: 1<br>Group 3: 1<br>Group 1: 0<br>Group 2: 1<br>Group 3: 1	saline<br>heparin 1mg/ml in saline<br>hirudin 1µg/ml in saline<br>saline<br>heparin 1mg/ml in saline<br>hirudin 1µg/ml in saline	intraoperatively	Group 1: 25<br>Group 2: 25<br>Group 3: 25<br>Group 1: 10<br>Group 2: 10<br>Group 3: 10	20%<br>16%<br>8%<br>40%<br>50%<br>20%	N.a.	N.a.
Ozbeck et al.	1994	Ear	Group 1: 1<br>Group 2: 1<br>Group 3: 0	Group 1: rhTPFI 40µg/ml<br>Group 2: heparin 50U/ml<br>Group 3: control buffer (saline)	intraoperatively, intraluminal	Group 1: 30<br>Group 2: 10<br>Group 3: 20	3%<br>40%<br>80%	N.a.	N.a.
Ramelli et al.	2019	cervical, femoral, tail	Group 1: 0<br>Group 2: 1	Group 1: saline<br>Group 2: UFH 5000UI/ml	intraoperatively, intraluminal	Group 1: 125<br>Group 2: 122	25%<br>13%	N.a.	N.a.
Reichel et al.	1987	A. femoralis	Group 1: 0<br>Group 2: 1<br>Group 3: 1<br>Group 4: 1<br>Group 5: 1<br>Group 6: 3<br>Group 7: 3	Group 1: Ringer solution<br>Group 2: heparin<br>Group 3: phentolamin<br>Group 4: urokinase<br>Group 5: TPA<br>Group 6: TPA, phentolamin, heparin<br>Group 7: heparin, urokinase, phentolamin	intraoperatively, intraluminal	Group 1: 40<br>Group 2: 40<br>Group 3: 20<br>Group 4: 20<br>Group 5: 40<br>Group 6: 40<br>Group 7: 20	90%<br>55%<br>65%<br>50%<br>85%<br>57.5%<br>45%	N.a.	N.a.
Ritter et al.	1998	A./V. femoralis	Group 1: 0<br>Group 2: 1<br>Group 3: 1	Group 1: Ringer solution<br>Group 2: 100U/kg UFH in 1ml of saline<br>Group 3: 1mg LMWH in 1ml of saline	intraoperatively	Group 1: 15<br>Group 2: 15<br>Group 3: 15	33.33%<br>13.33<br>0%	N.a.	N.a.
Rumbolo et al.	1991	A./V. femoralis	Group 1: 0<br>Group 2: 1<br>Group 3: 1	Group 1: Ringer solution<br>Group 2: Ringer with 20U/ml heparin<br>Group 3: Ringer with 500U/ml urokinase	intraoperatively, intraluminal	Group 1: 16<br>Group 2: 16<br>Group 3: 16	27%<br>33%<br>43%	N.a.	N.a.
Sinclair	1980	A./V. femoralis	Group 1: 1<br>Group 2a: 0<br>Group 2b: 1<br>Group 3: 1	Group 1: heparinised saline irrigation<br>Group 2a: saline irrigation and saline SC<br>Group 2b: saline irrigation and heparin SC<br>Group 3: heparinised saline irrigation	intraoperatively<br>pre-, intra- and postoperatively<br>pre-, intra- and postoperatively<br>intraoperatively	Group 1: 10<br>Group 2: 5<br>Group 3: 5<br>Group 4: 10	20%<br>100%<br>100%<br>30%	N.a.	N.a.

Vlastou et al.	1983	Ear replantation	Group 1: 1<br>Group 2: 0	Group 1: heparin 1000UI IV<br>Group 2: none	Intraoperatively	Group 1: 27<br>Group 2: 27	26%<br>66%	3.70%<br>N.a.	N.a.
Wallmichrath et al.	2015	Groin to Neck	Group 1: 0<br>Group 2: 0<br>Group 3: 1<br>Group 4: 1	Group 1: None<br>Group 2: 1ml Ringer over 10min<br>Group 3: 1ml heparin (100IU/kg) over 10min<br>Group 4: 1ml rtPA (2mg/kg) over 10min	Intraoperatively, intra-arterial	Group 1: 10<br>Group 2: 10<br>Group 3: 10<br>Group 4: 10	90%<br>90%<br>20%<br>40%	N.a.	N.a.
Wallmichrath et al.	2014	Groin to Neck	Group 1: 0<br>Group 2: 1	Group 1: 1ml Ringer<br>Group 2: 1ml Antithrombin (50UI/kg)	Intraoperatively, intra-arterial over 10min	Group 1: 11<br>Group 2: 11	81.81%<br>27%	N.a.	N.a.
Wolfort et al.	1992	A./V. femoralis	Group 1: 0<br>Group 2: 1	Group 1: 17cc/kg saline IV<br>Group 2: 1gm dextran 70	30min preoperatively and every 8h for 72h postoperatively	Group 1: 8<br>Group 2: 7	75%<br>0%	N.a.	N.a.
Yates et al.	2002	A./V. femoralis	Group 1: 0<br>Group 2: 1<br>Group 3: 1<br>Group 4: 2	Group 1: Ringer<br>Group 2: tirofiban (50µg/ml),<br>Group 3: heparin (100U/ml),<br>Group 4: heparin (100U/ml) and tirofiban (50g/ml)	intraoperatively, intraluminal	Group 1: 20<br>Group 2: 22<br>Group 3: 10<br>Group 4: 10	90% 20<br>41% 22<br>80% 10<br>20% 10	N.a.	N.a.
